# Assessing the landscape of initiatives to improve CKD early diagnosis and treatment

**DOI:** 10.1186/s12882-025-04678-z

**Published:** 2025-12-12

**Authors:** David Walters, David Bariau, Aurélien Bisquerra, Mikka Cabral, Diane Deville, Juliane Meyerhoff

**Affiliations:** 1 Boehringer Ingelheim International GmbH, Ingelheim am Rhein, Germany; 2 Alcimed GmbH, Cologne, Germany

**Keywords:** Chronic kidney disease, eGFR, Diagnosis, Medical education, Primary care, uACR

## Abstract

**Background:**

Although early detection of chronic kidney disease (CKD) and intervention can slow disease progression and reduce associated cardiovascular risks, systematic strategies for CKD screening and diagnosis remain inconsistent, with limited adoption of best practices across healthcare systems. This study aimed to assess the landscape of initiatives designed to improve early CKD diagnosis and treatment.

**Methods:**

A structured search was conducted between February and April 2024 to identify patient awareness, policy, and medical/non-medical education initiatives related to CKD early diagnosis and treatment developed by various stakeholders, including medical societies, patient organizations, pharmaceutical and medical technology companies, and government agencies. The search covered the United States, Germany, China, Japan, and global- or European-level programs. Initiatives were categorized based on their target audience, approach, and expected feasibility and impact. Expert interviews (*N* = 11) with primary care physicians (PCPs), nephrologists, and internists were conducted to evaluate awareness, perceived effectiveness, and challenges in CKD diagnosis and treatment. Medical education initiatives targeting healthcare providers (HCPs) were further analyzed to determine key gaps, and an impact/feasibility assessment was performed.

**Results:**

A total of 218 initiatives were identified, with the highest number in the United States, followed by China, Japan, and Germany. Most initiatives focused on patient and HCP education, with a significant portion led by pharmaceutical or MedTech companies and kidney foundations. However, awareness of these initiatives was low among PCPs. Five key gaps were identified among medical awareness initiatives: (1) lack of metric-driven goals, (2) limited peer-to-peer engagement in initiative design, (3) insufficient emphasis on serious outcome prevention, (4) inadequate targeting of high-risk populations such as patients with type 2 diabetes and hypertension, and (5) a general lack of PCP-focused interventions.

**Conclusions:**

Despite numerous educational initiatives aimed at improving CKD early diagnosis and treatment, gaps in implementation and awareness persist, particularly among PCPs. Addressing these gaps through data-driven goal setting, peer-to-peer engagement, and targeted interventions for high-risk populations could enhance the effectiveness of future programs. A more coordinated approach among stakeholders may help optimize CKD screening and management, ultimately improving patient outcomes and reducing the global burden of kidney disease.

**Supplementary information:**

The online version contains supplementary material available at 10.1186/s12882-025-04678-z.

## Introduction

Chronic kidney disease (CKD) represents a significant and growing public health concern, affecting more than 1 in 7 adults in the United States and 1 in 10 of the adult population globally [1]. Despite its prevalence, CKD remains largely undetected in its early stages, with approximately 90% of affected individuals unaware of their condition [1]. In the international retrospective observational study, REVEAL-CKD, the prevalence of undiagnosed stage 3 CKD ranged from 61.6% (USA) to 95.5% (France) [2]. Data from a German study found that guideline-recommended CKD screening was rarely carried out, even in patients with clear risk factors, with almost none receiving the recommended albuminuria test for early kidney damage [3]. This low rate of diagnosis is particularly concerning because the disease often progresses silently, typically manifesting symptoms only in advanced stages despite conferring increased risk of cardiovascular (CV) events; even early disease stages (with estimated glomerular filtrate rate [eGFR] > 60 mL/min/1.73 m^3^ but urinary albumin-to-creatinine ratio [uACR] ≥30 mg/g) are associated with markedly elevated risk for CV events, including death [4].

A delayed diagnosis of CKD is associated with significantly elevated risks of various serious outcomes including CKD progression to stage 4/5 CKD, kidney failure, myocardial infarction, stroke, and hospitalization for heart failure [5]. Multiple factors contribute to this diagnostic delay. These include limited awareness among both patients and healthcare professionals (HCPs); lack of standardized care protocols or clear guidance recommendations for screening specific populations; limited access to screening facilities; and limitations on reimbursement for laboratory testing, leading to subsequent underutilization of uACR screening in at-risk groups [6, 7]. Evidence-based interventions are available that can slow CKD progression, reduce CV risk, and have been shown to improve patient outcomes, and evidence strongly supports the benefits of early detection to enable timely intervention [8–11]. Late diagnosis and incomplete, infrequent, or unactioned laboratory values when screening people at risk of CKD result in subsequent delayed diagnosis and, therefore, represent a missed opportunity for early intervention and improving patient outcomes.

Early detection and intervention in CKD is not a novel concept [12], but its implementation remains inconsistent. Current national and international guidelines recommend early diagnosis and treatment, particularly as nephroprotective treatments can improve both kidney and CV prognoses [9]. However, there is no universally accepted systematic strategy for CKD diagnosis and treatment, nor consensus on optimal screening approaches; although recommendations often advocate for screening of at-risk patient populations the optimal frequency of screening can be unclear, especially as this differs among patient groups. A recent systematic review that examined the literature for CKD screening programs in the United States and other English-speaking countries revealed concerning gaps in implementation [13]. The authors found a low prevalence of CKD screening among at-risk patients in primary care settings, though higher rates of screening were observed in indigenous populations (91%) and community outreach settings (93%). Importantly, most at-risk patients received incomplete screening that did not align with Kidney Disease: Improving Global Outcomes (KDIGO) guidelines [4], and follow-up testing was either infrequent or unreported in most studies.

Participants at a 2019 KDIGO controversies conference on “Early Identification and Intervention in CKD” unanimously agreed that the evidence supports a systematic approach to screening for and treating people with CKD [10]. Given the availability of accurate and low-cost diagnostic tests, attendees endorsed a broad and proactive plan for CKD screening, risk stratification, and treatment to help reduce the global burden of kidney disease. Despite the availability of effective and affordable treatments to slow disease progression and reduce CV risk, accurate diagnosis and staging are essential for effective treatment implementation. Therefore, screening is recommended for at-risk populations, such as people with hypertension, diabetes, or CV disease, with screening frequency and timing determined according to individual risk profiles and preferences. Screening requires assessment of both eGFR and albuminuria [9, 10].

Separate initiatives have been led by multiple medical societies, pharmaceutical companies, and patient associations, resulting in a lack of clarity for HCPs and especially primary care physicians (PCPs). This analysis is intended to provide a broad landscape assessment of existing initiatives to improve CKD early diagnosis and treatment, to identify potential success factors for implementation and deployment among the initiatives identified, and to pinpoint any specific gaps that could potentially be addressed by new initiatives.

## Methods

We performed a methodological search to identify initiatives to improve CKD early diagnosis and treatment, before characterizing the initiatives identified. A further analysis of initiatives targeted to HCPs/PCPs was performed to identify gaps and key success factors.

### Identifying and categorizing initiatives

Searches were performed between February and April 2024 to identify educational initiatives, initiatives focused on monitoring key performance indicators related to reducing the rates of CKD, laboratory results reporting, or policy-oriented initiatives whose aim was to increase early CKD diagnosis and treatment. Initiatives could be conducted by medical societies, HCP associations, patient associations, pharmaceutical and MedTech companies, insurance companies, and any other relevant stakeholders (see Supplemental file 1). These initiatives could include programs, campaigns, tools, or actions aimed at increasing early diagnosis and treatment of CKD, and where this was only part of a broader initiative these were also included; real-world evidence studies and initiatives focusing on the treatment of CKD were excluded. Searches were limited to initiatives in the USA, Germany, China, and Japan, and those at the global or European level. Searches for each region were performed in the local language and in English, except for China where searches were done in Mandarin Chinese only. Key stakeholders relevant to CKD—including national, regional, and global medical societies and patient advocacy groups, pharmaceutical companies, MedTech companies, HCP associations, medical societies, insurance providers, and government groups—were identified. For each stakeholder, a search was done on relevant websites and social media channels for initiatives (Supplemental file 2). Additional searches were performed across PubMed, social media (YouTube, Instagram, X, and regional platforms such as Baidu), and national specialist magazines such as *Deutsches Ärzteblatt*.

External experts (11 in total; 6 in the USA, 5 in Germany; 2 additional experts were contacted but chose not to participate), including PCPs, nephrologists and internists, were selected for interview based on their role within leading professional associations to ensure they had knowledge of the wider landscape beyond their clinical own practice. Nephrologists and internists were also selected based on their knowledge and expertise in the screening and diagnosis of CKD. Interviews were developed for this study; details are available in the interview guide (Supplemental file 3).

For each initiative identified, the following information was collected: detailed information around the sponsor/developer; date and target area of initiative launch; deployment channel; primary and secondary target audiences; aim and a qualitative assessment of expected impact (developed in conjunction with feedback from the expert interviews). All available information, including the expert interviews, was leveraged to assess the level of knowledge and awareness of the various initiatives among HCPs, and to identify any “pain points” (problems or issues) as perceived by PCPs.

Key success factors and educational gaps were qualitatively assessed for initiatives in Germany and the USA only, with gaps and success factors hypothesized based on the interviews and refined following feedback from a panel of four experts with experience in early screening initiatives across various therapeutic areas. The refined key success factors and educational gaps were then confirmed against the list of initiatives identified. We then assessed what proportion of the educational initiatives identified addressed one or more of the educational gaps. An impact score (5 = most impactful, 1 = least impactful) was subjectively assigned following discussion with external stakeholders during the interviews to denote the potential impact each initiative could have on increasing the screening and diagnosis rates of early CKD based on the feasibility of each gap (i.e. the ease in which an initiative could be developed and deployed) versus its relative impact (i.e. the extent to which each gap could impact the rates of screening and diagnosis of early CKD).

## Results

**Fig. 1:**
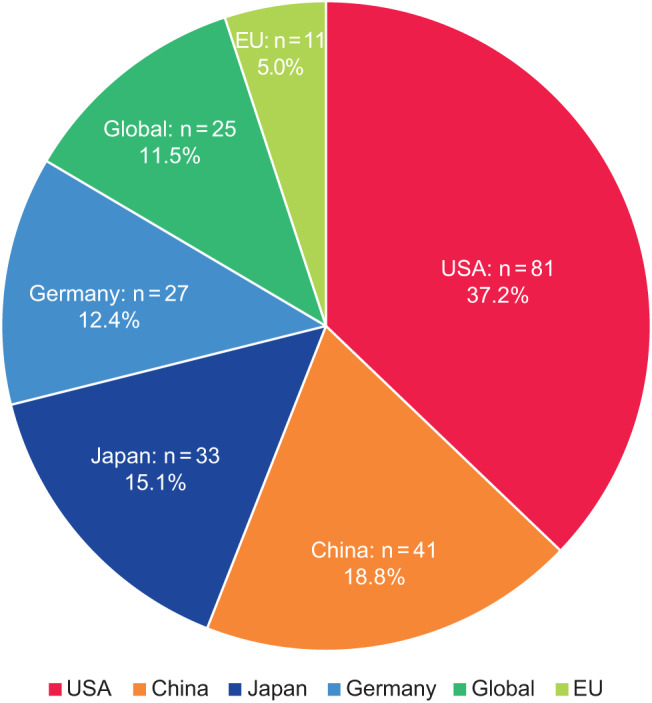
Initiatives (*N* = 218) by region/country. EU, European Union; USA, United States of America

**Fig. 2:**
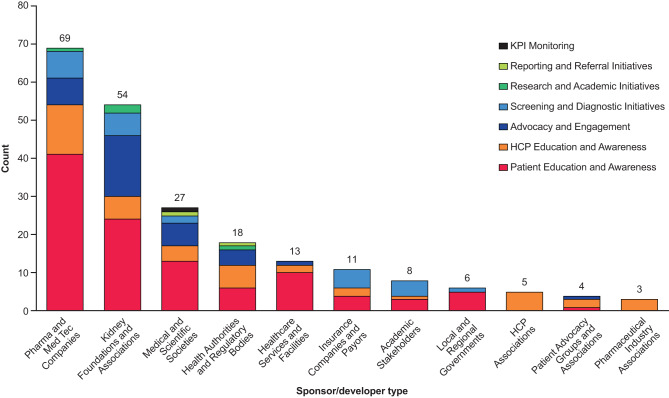
Initiatives by sponsor type (*N* = 218). HCP, healthcare professional; KPI, key performance indicator

**Fig. 3:**
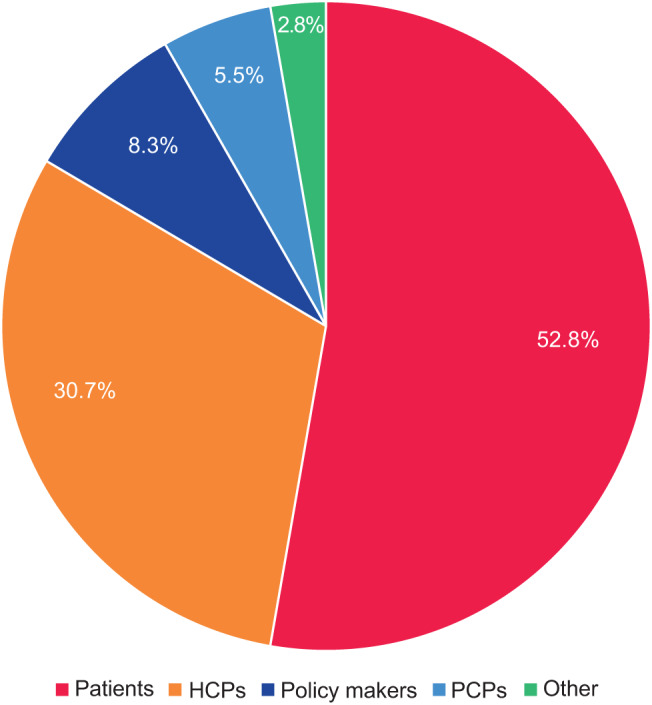
Initiatives (*N* = 218) by primary target audience. “other” includes one each of the following categories: Health systems; Industry players; Laboratories; Nurses; Payors; Researchers. HCPs, healthcare professionals; PCPs, primary care practitioners

In total 218 initiatives were identified, with almost twice as many based in the USA (*n* = 81) as the next country, China (*n* = 41), followed by Japan (*n* = 33) and Germany (*n* = 27; Fig. 1). A further 11 EU-wide initiatives were identified, along with 25 global initiatives. Patient and HCP education and awareness initiatives were the most common types of initiative identified, mostly developed or led by pharmaceutical/MedTech companies or kidney foundations and associations (Fig. 2). Over one-fifth of the initiatives identified were informational awareness webpages (22.0%, 48/218); the next most common were calls to action (6.9%, 15/218), screening initiatives (6.4%, 14/218), and patient education sessions (6.0%, 13/218) (Table 1). The majority of initiatives were targeted toward patients (115/218, 52.8%), followed by HCPs (67/218, 30.7%) and policymakers (18/218, 8.3%); only 12 (5.5%) were found to explicitly target PCPs, as opposed to HCPs in general (Fig. 3).

**Table 1:** Initiatives by subcategory (*N* = 218)

Initiative subcategory	Count
Awareness webpage	48
Call for action	15
Screening	14
Patient education session	13
Conference/Meeting	10
Campaign	10
Project(s)	9
Video	9
Educational materials	8
Guide	7
Guidelines	7
Toolkit	6
Training	6
Quiz	5
Risk calculator	5
Test kits	4
Webinar	4
Article	4
Research	4
HCP newsletter	4
Policy	4
Press release	3
Radio	2
White Paper	2
Referral encouragement	2
Insurance plan	2
Forum	2
Digital platform	2
Manifesto	1
KPI definition and progress tracking	1
Survey	1
Report	1
Sponsorship/Support	1
Review	1
Expo	1

See Supplemental file 1 for definitions. HCP, healthcare provider; KPI, key performance indicator

Of the initiatives identified, 79 had a primary target audience of HCPs or PCPs; patients were often considered a secondary target—such as educational materials for HCPs to download and distribute to their patients, or the inclusion of patient-facing wepages. Despite the high number of these HCP-targeted initiatives identified in Germany and the USA, feedback and self-assessment during interviews revealed a generally low level of awareness among PCPs regarding these initiatives, and a medium level of awareness among nephrologists. Various pain points arise in the diagnosis and treatment of early CKD at the PCP level, including a generally low confidence in their ability to treat and make appropriate referrals, lack of time and resources, insurance-related issues, and a general lack of staying up to date with new developments in the field of CKD (Table 2). However, certain existing initiatives were assessed as useful by US HCPs in circumventing the key pain points, such as the KDIGO heat map, cross-disciplinary consultations between PCPs and nephrologists, and awareness campaigns targeting PCPs. Although many of the issues identified were beyond the control of those developing new initiatives, the following key factors were mentioned by HCPs in the USA and Germany as contributing to the successful development and deployment of new initiatives.

- **National design**: Initiatives should be tailored toward a country’s specific healthcare and reimbursement system, to ensure full functionality in the context in which it is deployed.
- **PCP focus:** Initiatives should mainly focus on the further education of PCPs regarding screening, treatment, and referral practices around CKD, to establish earlier diagnosis and decrease referral rates.
- **Peer leadership:** Although initiatives can be funded by pharmaceutical and MedTech companies, to build trust among HCPs it is important that they are presented and advocated for by medical peers and/or medical societies.
- **Easy data collection:** Initiatives should aim to advocate for practices that allow easy and cost-effective collection of data, which would motivate HCPs to deploy an initiative on a large-scale basis.

**Table 2:** PCP “pain points” that arise in the diagnosis and treatment of early CKD

**Lack of treatment confidence and appropriate referral** At the PCP level, there is a general lack of knowledge and confidence to treat patients due to an unawareness regarding the right therapies to prescribe associated to specific values coming out of the urinalysis. PCPs mention that they are typically not aware or not fully aware of their role in the CKD treatment pathway, and what is expected of them regarding CKD treatment. *“The main issue with CKD at the PCP level is that we are not always clear until when our role extends to, and when we should handover to the specialist … As well as not being fully confident in the treatment protocol.”*** PCP, member of American College of Physicians, USA** *“PCPs refer when they detect CKD, even if at stage 2–3, probably because they are not confident in their treatment capability at their level.”*** Nephrologist, member of German Society of Nephrology, Germany**
**Lack of time and resources** PCPs report having an overwhelming number of patients, many of them presenting risk factors for cardiovascular disease and T2D, thus inherently at risk for early CKD. Due to the sheer number of patients, PCPs can get desensitized to abnormal GFR values and sometimes do not have the capacity to do follow-up uACR tests on all patients who fall into the high-risk category, with a limited sense of urgency when it comes to uACR testing. Additionally, patients can be reluctant to attend the follow-ups as they do not experience physical symptoms of CKD, thus not prioritizing the consultations. *“We do not have a budget to screen for early CKD. A PCP is allowed 1,60 EUR per patient for lab testing. If we conduct lab testing for a patient which costs for instance 10 EUR, then roughly the next 10 patients cannot have lab testing done or the practice loses money. The same goes for treatment.” * **PCP, board member of regional Primary Care Physicians Association and PCP in diabetes clinic, Germany**
**Screening and prescription limitation by health insurance** German PCPs have a defined envelope for performing lab testing for each patient, which does not allow for thorough screening. Consequently, PCPs refer the patient to a specialist if they see the need for specific medications or follow-up tests. Moreover, PCPs report issues in prescribing newer, more expensive medications such as finerenone or SGLT2 inhibitors, as the healthcare system limits the monetary prescription costs of PCPs and penalizes them if they surpass it, leaving them reluctant to prescribe such medications over concerns of surpassing the allotted quota. In the United States, PCPs face similar issues, with insurance companies challenging the prescription of finerenone and SGLT2 inhibitors for patients with early CKD, only agreeing to reimbursement if strongly motivated (which typically only arises in later stages of CKD). Thus, PCPs generally avoid prescribing such medications to early-stage CKD patients. *“We try to keep up to date with all the new developments in general, and of course CKD is a large part of our daily practice, however the field evolves extremely fast and we don’t often get the time to read and be updated with all the new data on the novel medications … which also means that we might not be fully aware of the medical benefits of some of the new drugs.”*** PCP, member of associations for care co-ordination and public health in large university hospital, USA** *“Testing for CKD is part of our daily practice, but at the same time we may not be up-to-date with all the benefits of a more thorough testing, and thus we don’t accord it as much importance as we perhaps should.”*** PCP, member of American College of Physicians and member of Internal Medicine and Pediatrics associations, USA**
**Difficulty keeping up with innovations in CKD** CKD is a fast-evolving, dynamic field. Given their time limitations, it is hard for PCPs to keep track of new developments and treatments. They are not always aware of improvements in existing drugs, novel drug releases and the evidence behind them, resulting in a fragmented view on the holistic treatment options and the respective efficacy profile of newer drugs. Consequently, PCPs might not have the right knowledge to prescribe the most appropriate medications.

Note: Ranked by frequency of mentions by interviewees, from the most to the least mentioned

CKD, chronic kidney disease; GFR, glomerular filtration rate; PCP, primary care physician; SGLT, sodium/glucose cotransporter; T2D, type 2 diabetes; uACR, urinary albumin-to-creatinine ratio

Two additional key success factors specific to the US healthcare system were also prominent regarding initiatives targeting early CKD screening and diagnosis.

- **Compatibility with electronic medical records:** Outcomes data obtained through the initiatives should be compatible with local EMR systems, so that success of the initiative can be clearly quantified.
- **Financial incentives:** Adherence increases when PCPs are financially incentivized to participate; the success rate of such initiatives, such as in diabetes screening, is historically higher in the USA.

Around 9% (*n* = 20) of initiatives identified targeted a direct impact on the screening and diagnosis of CKD (e.g. by offering free screening services to patients; putting patients directly in contact with HCPs; or encouraging PCPs to recognize, treat, or refer early CKD patients). Overall, an emotional response was targeted by around 5% of initiatives (*n* = 11), including four developed in China.

Following review of the educational initiatives targeting HCPs and/or PCPs, five main gaps were identified:

1. **A lack of a peer-to-peer approach in the design of the initiative.** Most initiatives aimed at PCPs were developed by government bodies, nephrology associations, and pharmaceutical companies alone, with only 4 out of the 79 identified initiatives (~5%) involving PCPs and PCP associations in design and promotion. These initiatives utilized various channels but did focus on sharing PCP resources (e.g. diagnosis guides, patient counseling resources, information brochures).
2. **A lack of definition and tracking of metric targets to convince PCPs.** Only 6 of the 79 initiatives (~8%)—1 in Japan, 1 in the USA, and 4 Boehringer Ingelheim initiatives—set clear goals and trackable metrics allowing stakeholders to see the progress in the way an initiative improves early CKD screening and diagnosis rates. Both external initiatives were strategic projects focused on HCP awareness and resources.
3. **A lack of focus on serious outcome prevention.** Although some mentioned the need to slow disease progression, only 8 of the 79 initiatives described in detail the patient outcomes that were being avoided; specifically, CV outcomes and dialysis. Around 10% of initiatives fill this gap, mostly websites focused on HCP awareness.
4. **A lack of focus on patients with type 2 diabetes (T2D) or hypertension.** Only 9 of the 79 initiatives specifically targeted patients with T2D and other known at-risk populations, such as people with hypertension, with many describing “at-risk” patients without further specification. Around 11% of initiatives filled this gap, mostly websites focused on HCP awareness.
5. **A lack of focus on PCPs.** The majority of initiatives (68/79; ~86%) targeted HCPs in general, rather than directly addressing the type of HCP who sees the most patients at risk for CKD. Around 14% of initiatives filled this gap, primarily websites focused on HCP resources.

Initiatives that addressed the five gaps varied in relative feasibility and impact, with those filling gaps 3, 4, and 5 being relatively high in feasibility and medium to high in impact. Six of the initiatives identified addressed two of the gaps, while one addressed four (Supplemental file 4).

## Discussion

This methodological analysis looked at the landscape of existing initiatives to improve CKD early diagnosis and treatment, to pinpoint any specific gaps in those targeted to HCPs/PCPs that could potentially be addressed by new initiatives. We identified 79 initiatives that targeted HCPs/PCPs, and amongst these we identified five gaps that were not widely addressed by the current range of initiatives. Based on these findings, our recommendations for future initiatives to address these gaps are as follows:

### Peer-to-peer design approach

Targeting PCPs via a peer-to-peer approach may reassure PCPs that their concerns have been considered and may help to increase their involvement with implementing screening. Greater involvement of PCPs in initiative development through peer-to-peer approaches, ambassador programs, or PCP-led training, along with professional society collaborations, could improve adoption rates by ensuring that solutions are both practical and aligned with real-world clinical workflows.

### Definition and tracking of metric targets

The absence of well-defined success metrics in most initiatives makes progress difficult to quantify, reducing accountability and potentially limiting long-term engagement from stakeholders. Establishing clear, measurable objectives—such as increased screening rates or improved guideline adherence—and putting a system in place to collect the relevant data for tracking would show HCPs exactly how many patient lives are improved. Demonstrating the effectiveness of the initiative could also help to secure ongoing funding and resource allocation, justifying HCP/PCPs’ efforts to increase screening and encouraging them to continue. By monitoring defined metrics, initiative developers can identify areas of underperformance, allowing them to make educated adjustments to improve the initiative, build a business case for ongoing implementation and subsequent phases, or reallocate resources as required.

### A focus on serious outcome prevention

Increasing communications on the serious impact of late-stage CKD on patients and their quality of life can help HCPs and patients appreciate the importance of future initiatives. Existing initiatives often fail to communicate the serious long-term consequences of CKD progression, including CV events and dialysis dependence. Notably, the CV risks associated with CKD, even in its early stages, are not currently communicated adequately to PCPs or patients. Future initiatives should utilize patient stories and/or PCP experiences to emphasize the burden that delayed CKD diagnosis can cause. More effective messaging emphasizing these risks and the availability of treatment options that can slow disease progression and improve patient outcomes, particularly within PCP-targeted programs, could encourage earlier intervention. Similarly, expanding initiatives to more explicitly target high-risk populations, including individuals with hypertension and T2D, could enhance screening efficiency and diagnostic yield.

### A focus on patients with T2D, hypertension, or CV disease

Future initiatives should target patients with T2D and other specific at-risk groups, such as people with hypertension and CV diseases. Prioritizing such common at-risk groups will help to ensure that an increase in screening leads to an increase in diagnosis, optimizing the value of screening efforts.

### PCPS and internists should be the primary target of educational initiatives

Although numerous initiatives have been developed globally, their overall effectiveness remains constrained by limited awareness, lack of systematic coordination, and insufficient engagement with PCPs. Given that PCPs and internists are best positioned to screen, diagnose, and manage patients with early-stage CKD [14], the limited targeting of this group represents a major missed opportunity. Integrating CKD awareness and management strategies into PCP training and practice guidelines, along with providing decision-support tools embedded in electronic medical records, could significantly improve early diagnosis rates. Other HCPs could also be targeted. For example, diabetologists and endocrinologists order blood tests and conduct regular follow-ups with at-risk patients, but they were not specifically targeted by any observed initiatives. Similarly, although cardiologists are more likely to be involved in care for CV diseases, they should be included in holistic assessment and prevention management strategies, given that CKD contributes substantially to CV risk.

Ultimately, a more coordinated, data-driven, and PCP-centered approach is needed to close these gaps and maximize the impact of early CKD diagnosis and treatment initiatives. However, in terms of increasing early diagnosis and treatment of CKD, the success of any initiative depends on more than just closing the above-mentioned gaps; education and awareness are not the only issues that need to be addressed. There are likely to be multiple “environmental” factors such as reimbursement, policies and guidelines, and positive or negative incentives that will impact on the uptake of any initiatives. Furthermore, developing a website requires additional promotion to the target audiences to raise awareness of the resource.

The REVEAL trial showed us the blind spots in CKD diagnosis, highlighting the shockingly high proportion of CKD that remains undiagnosed and underscoring the need to improve rates of early diagnosis and screening [2]. Initiatives to improve screening rates based on simple principles, such as the ABCDE approach, aim to empower individuals and HCPs to assess risk of kidney disease [15]. However, it must be remembered that screening is only the first, albeit vital, step in the treatment journey; even where CKD has been diagnosed, substantial gaps in care remain [16].

## Limitations

This search was performed between February and April 2024, and only included initiatives that had publicly available information online during this period. Some initiatives may not have been captured if they were no longer visible online at that time, or if they were active but not yet sharing data. For most initiatives, specific metrics (e.g. number of visitors to a website, downloads) to measure the impact of the initiatives or outcomes were unavailable or could not be assessed. No attempt was made to determine what additional activities sponsors could have performed to increase the reach and impact of individual initiatives (e.g. presence at congresses, emails, social media posts). The countries included in this assessment were chosen for their well-developed healthcare systems and sizeable populations and the selection was intended to provide a balance between Western and Eastern representation, as there may also be cultural differences in how disease state education is conducted. Evidence suggests that there are significant disparities in the organization, infrastructure, and delivery of services for CKD in different countries [17], and the difference between models of care may have influenced the way initiatives are set up. Thus, the relevance of our findings to other countries with differing healthcare systems is uncertain. Interviews to assess awareness were only conducted with a limited sample of individuals who were selected for their expected knowledge of the field, so general awareness across the wider HCP environment is likely to be lower.

## Conclusions

The findings of this landscape analysis highlight the extensive efforts undertaken by several types of stakeholder to improve early CKD diagnosis and treatment, as well as pointing to substantial gaps in their implementation. We identified five primary gaps within existing educational initiatives; their effectiveness might be improved by modifying factors such as the use of metrics and measurable objectives, engagement of PCPs in peer-to-peer approaches, communication of long-term consequences of CKD and opportunities for early intervention, and a focus on common populations at risk for CKD (e.g. diabetes and hypertension). Multiple stakeholder collaboration—including HCPs, policymakers, pharmaceutical and MedTech companies, and patient advocacy groups—will be crucial to optimizing CKD screening and management strategies. By addressing current deficiencies and leveraging evidence-based best practices, future initiatives can contribute to reducing CKD-related morbidity and mortality, helping to alleviate the global burden of this disease.

## Electronic supplementary material

Below is the link to the electronic supplementary material.

Supplementary Material 1

Supplementary Material 2

Supplementary Material 3

Supplementary Material 4

## Data Availability

The datasets coming from desk research used and/or analyzed during the current study are available from the corresponding author on reasonable request. The data coming from interviews and supporting the findings of this study are available from Alcimed GmbH but restrictions apply to the availability of these data, which were used under license for the current study, and so are not publicly available. Data are available from the authors upon reasonable request and with permission of Alcimed GmbH.

## Abbreviations

CKD: Chronic kidney disease
CV: Cardiovascular
eGFR: Estimated glomerular filtrate rate
EMR: Electronic medical record
HCP: Healthcare provider
KDIGO: Kidney Disease: Improving Global Outcomes
PCP: Primary care physician
T2D: Type 2 diabetes
uACR: Urinary albumin-to-creatinine ratio
