# Supplementary material for: Assessing the landscape of initiatives to improve CKD early diagnosis and treatment
Source: BMC Nephrol. 2025 Dec 12;27:50. doi: 10.1186/s12882-025-04678-z (PMC12817422; doi:10.1186/s12882-025-04678-z)
Supplement: Supplementary file 1 — Supplementary Material 1 [file 12882_2025_4678_MOESM1_ESM.pdf]

**Search strategy**

Every stakeholder in the 4 countries involved with CKD were investigated with the following search terms including but not limited to:

> STAKEHOLDER NAME AND ("chronic kidney disease" OR CKD) + (campaign OR initiative OR action OR awareness OR diagnosis OR screening OR early screening OR patient awareness OR training OR webinar OR early detection OR conference)

This search was done on a stakeholder's homepage, press releases, social media accounts (YouTube, X, Instagram, LinkedIn etc.) and search engines in local language.

## Additional file 1. List of stakeholders for initial search

Additional stakeholders were identified during the search and from expert interviews.

- Abbott
- AstraZeneca
- Bayer
- Baxter
- American Association of Kidney Patients
- American Heart Association
- American Kidney Fund
- American Society of Nephrology
- Boehringer Ingelheim
- Bundesverband Niere (Federal Kidney Association)
- Centers for Medicare and Medicaid
- Chinese Society of Nephrology
- Deutsche Gesellschaft für Allgemeinmedizin und Familienmedizin (German Society for General Practice and Family Medicine)
- Deutsche Gesellschaft für Nephrologie (German Society for Nephrology)
- Deutschen Diabetes Gesellschaft (German Diabetes Society)
- Deutsche Nierenstiftung (Germany Kidney Foundation)
- Deutscher Hausärzteverband (German General Practitioners' Association)
- European Alliance for Cardiovascular Health
- European Kidney Health Alliance
- European Kidney Patients' Federation
- European Renal Association
- Fresenius Kidney Care
- German Foundation for Chronic Diseases
- International Society of Nephrology
- Japan Kidney Association
- Japanese National Health Insurance
- Japanese Society of Nephrology
- KDIGO
- Johnson & Johnson
- Kyowa Kirin
- Medical schools
- Medtronic National Clinical Research Centre for Kidney Disease in China
- National Health Commission of the People's Republic of China
- National Institute of Diabetes and Digestive and Kidney Diseases
- National Institutes of Health
- National Kidney Foundation
- Otsuka Pharmaceuticals
- Renal Physicians Association
- Roche Diagnostics
- Siemens Healthineers
- Statutory Health Insurance Companies in Germany (GKV)
- World Kidney Day
