# Supplementary material for: Assessing the landscape of initiatives to improve CKD early diagnosis and treatment
Source: BMC Nephrol. 2025 Dec 12;27:50. doi: 10.1186/s12882-025-04678-z (PMC12817422; doi:10.1186/s12882-025-04678-z)
Supplement: Supplementary file 2 — Supplementary Material 2 [file 12882_2025_4678_MOESM2_ESM.pdf]

Additional file 2. Definitions of initiatives and categories

Definitions

- **EU-level initiative** – Aims to act at an EU level, rather than focusing on specific countries within the EU
- **Global-level initiative** – Developed on a global scale, without any country or region specificities
- **Direct initiative** – May have a direct impact on CKD screening, diagnosis and treatment by offering free screening services to patients, putting patients directly in contact with HCPs, or encouraging PCPs to recognize, treat or refer early CKD patients
- **Indirect initiative** – May not have a direct impact on CKD screening, diagnosis and treatment as it may not require immediate action or may not bring together at-risk populations and HCPs

Initiative categories

- **Patient education and awareness** – Aim to increase awareness of CKD among patient populations and increase education levels regarding the disease itself, as well as the benefits of early screening
- **HCP education and awareness –** Aim to increase the visibility of CKD among HCPs, as well as initiatives to improve HCP education around the screening, diagnosis and treatment of the disease
- **Advocacy and engagement** – Aim to improve the health policy, recommendations or current practices regarding CKD screening, diagnosis and treatment
- **KPI monitoring** – Projects that aim to track and monitor key performance indicators of initiatives aimed at improving the diagnosis and care of CKD
- **Screening and diagnostic initiatives** – Aim to offer screening services, increase access to screening or connect patients and HCPs to facilitate diagnosis
- **Reporting and referral initiatives** – Aim to improve the diagnosis of CKD via lab result reporting or by improving the referral of potential CKD patients to specialists
- **Research and academic initiatives** – Aim to promote research and academic endeavors in CKD screening and diagnosis

Initiative subcategories

- **Screening** – Free screenings, medical consultations etc
- **Test kits** – Development or distribution of CKD test kits
- **Referral encouragement** – Encouragement of PCPs to recognize and refer early CKD patients
- **Call to action** – Communications to regulatory stakeholders and policymakers to encourage policy recommendations to improve CKD care
- **Project(s)** – One or more projects, usually involving a combination of initiatives to tackle problems in CKD care
- **Policy** – Policy changes to improve CKD care
- **Toolkit** – A combination of materials (usually related to education, referral encouragement, patient communication etc)

- **Training** – Training modules for HCPs to improve CKD screening, diagnosis and/or care
- **Conference/Meeting** – Meetings bringing together key stakeholders
- **KPI definition and progress tracking** – Progress tracking for existing initiatives to increase screening, diagnosis and treatment of early CKD
- **Guidelines** – Regulatory guidelines for CKD care
- **Campaign** – Wide-reaching education and awareness materials on early CKD care
- **Insurance plan** – Insurance policies with specific features to improve CKD care
- **Patient education session** – In-person discussion or lectures to improve CKD education in the general public and at-risk populations
- **Webinar** – Online lecture or discussion on CKD care
- **Manifesto** – A document discussion an aspect of CKD care in detail with a wide scope
- **Survey** – A collection of responses from HCPs or patients on CKD care
- **Review** – Analysis of a wide range of topics regarding CKD care
- **Report** – A report of an analysis regarding CKD care
- **Risk calculator** – A tool to assist patients or HCPs in understand the potential risk of CKD in a patient
- **Research** – The intent or development of a method to analyze some aspect of CKD care
- **Guide** – Recommendations for best practices in CKD care
- **Educational materials** – Physical or virtual materials aimed at increasing the CKD education in HCPs or patients
- **Video** – A video on CKD care
- **Radio** – A radio broadcast on CKD care
- **White Paper** – An informational document to promote the features of a service or product related to CKD care
- **Forum** – A platform to engage in discussions related to CKD care
- **Digital platform** – A platform to receive information, interact with patients or HCPs, download resources etc related to CKD care
- **Expo** – A display of products and services related to CKD care
- **HCP newsletter** – A communication aimed at HCPs to share news related to CKD care
- **Article** – A written piece by an academic, HCP or known stakeholder on a topic related to CKD care
- **Quiz** – An interactive tool to gather information on a patient's knowledge of CKD
- **Press release** – A release of information on a new service or product or results of a trial related to CKD care
- **Awareness webpage** – An online webpage to disseminate information on CKD to a wide range of audiences
- **Sponsorship/Support –** The provision of support (financial or otherwise) to an existing campaign or project developed by a third-party entity
