# Supplementary material for: Assessing the landscape of initiatives to improve CKD early diagnosis and treatment
Source: BMC Nephrol. 2025 Dec 12;27:50. doi: 10.1186/s12882-025-04678-z (PMC12817422; doi:10.1186/s12882-025-04678-z)
Supplement: Supplementary file 3 — Supplementary Material 3 [file 12882_2025_4678_MOESM3_ESM.pdf]

Interviews

Interviewees were approached via email or online message. Following agreement to participate, 1-hour interviews were conducted at their place of work with only the interviewer and the interviewee present. Interviewees were informed of the interviewer and Alcimed's name, and what the shared aim of the project and intended use of data was. They were also told that the work was being done on behalf of a pharmaceutical company but the sponsor company was kept anonymous.

The interview guide was developed by the authors but was not pilot tested. No specific scientific framework was used for the analysis of interview insights. However, we used the exact same questions as the basis of each discussion (semi-guided interview). We then compared their answers, challenged their viewpoints between interviewees, and constructed our analysis/opinion based on the viewpoints of the different stakeholders.

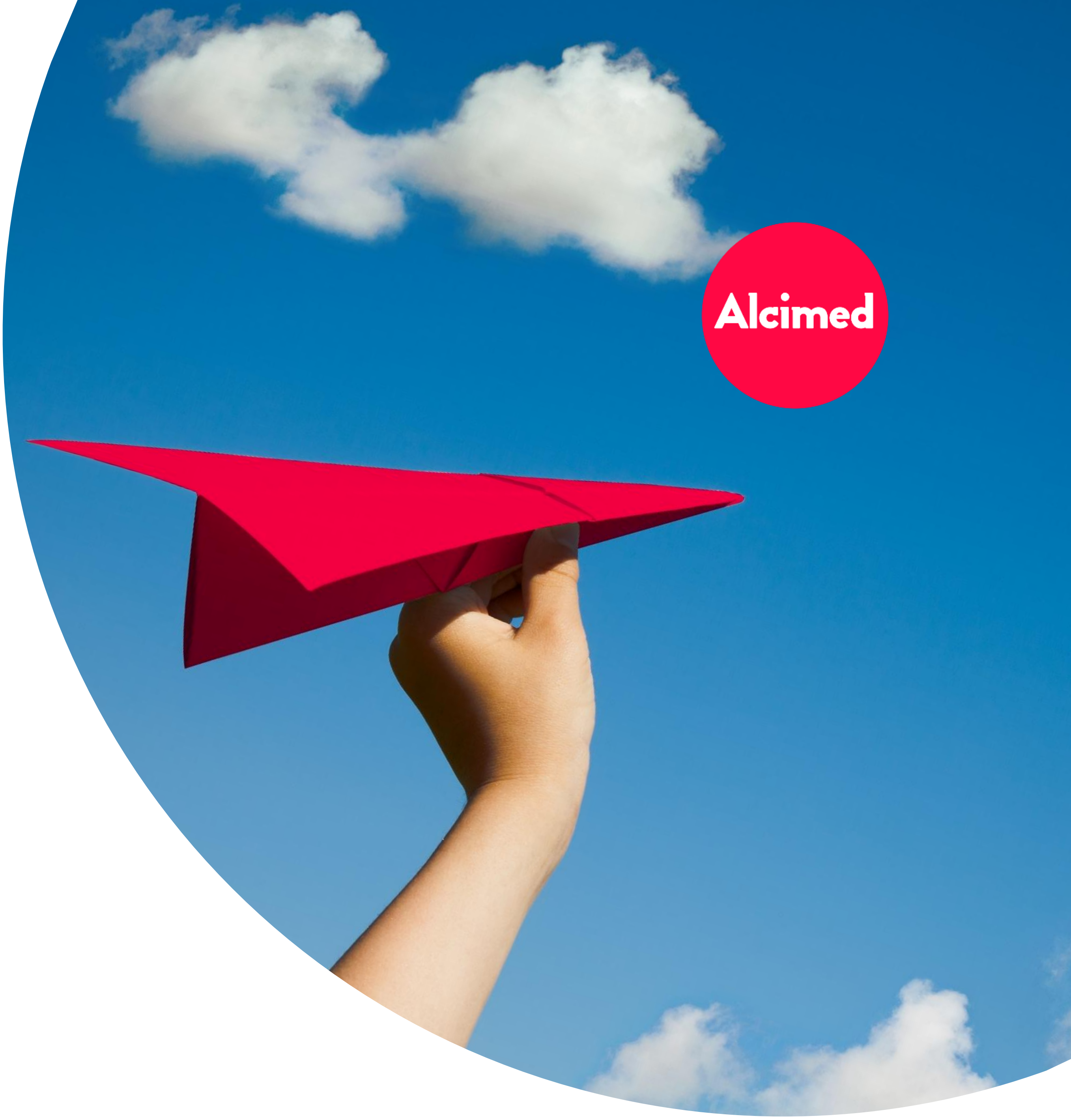

April 2024

Project Early CKD Interview Guide

Structure of the Discussion

Introduction

[Name], consultant in healthcare at Alcimed in Cologne, Germany - a consulting firm where we conduct explorations in the field of chronic kidney disease. Thank you so much for taking the time for this discussion.

As I briefly told you per e-mail, I am leading a project for my client, whose aim is to map initiatives aimed at increasing the screening and diagnosis rates of early CKD and to understand pain points of early CKD screening and the gaps in current initiatives to increase it. This is why I contacted you, as a/n [position and speciality].

[If asked] Our discussion is double-blinded and please let me know whenever you don’t wish to answer a given question.

Part I: Mapping Initiatives

1. Can you name the initiatives you are aware of which aim to increase the screening or diagnosis of CKD at a national or a global level (currently running or in development)?
2. For each initiative:
   a. Who is the target audience of the initiative (e.g., patients, PCP, specialist etc.)?
   b. What is the expected impact of the initiative?
   c. In your opinion, what are the strengths of the initiative?
   d. In your opinion, what are the weaknesses of the initiative?
   e. How successful is the initiative and how was this measured?

[If no initiatives are mentioned]

3. Why do you think that there are no initiatives that you are aware of with this aim to increase screening and diagnosis for early CKD?
4. Are the any other initiatives with the same aim for other disease states that you are aware of?

Part II: Gap Analysis & Pain Points

1. How are the guidelines to screen at-risk individuals followed?
2. In your opinion, what are the pain points associated with diagnosis and screening of early CKD?
   a. From the patient's perspective?
   b. From the primary care physician’s perspective?
   c. From the specialist's perspective (nephrologist, diabetologist, endocrinologist)?
   d. From the payors perspective?
   e. From the policymaker’s and regulatory stakeholder’s perspective?
3. How successful are the current initiatives in addressing these pains points and increasing the rate of diagnosis of early CKD?
4. Where do you believe there to be gaps in the initiatives currently deployed?
   a. Within the initiatives themselves (content, target audience, coverage)?
   b. Within the translation from initiative into practice? How can this be optimized?
5. Which types of initiatives do you think are successful in addressing these pain points? Why? Can you name examples?
   a. *[If multiple successful examples named]* How can these successful initiatives be brought together in a more useful and accessible way?
   b. Who do you think should be the stakeholder to initiate this?

6. Which types are initiatives are not successful? Why?
7. Which stakeholders involved in the screening and diagnosis of early CKD are not engaged or targeted enough in the initiatives currently deployed?
8. Who are the most important stakeholders to target for an initiative to be successful?
9. What is the level of awareness of primary care physicians of the need to increase early CKD screenings in [*country*]?
10. In an ideal world and with unlimited budget, what initiative(s) would you launch?

Part III: Key Performance Indicators and Impact Measurement

1. How would you measure the success of an initiative to increase early CKD screening and diagnosis?
    a. What are the key targets to be met and how to measure them?
2. When developing a new initiative, what needs to be considered before launch to ensure that the success of the initiative can be measured after the launch?

Part V: Closing

1. Thank interviewee.
2. Explain next steps.
3. Ask about willingness to be contacted again for follow-up questions.
