# Supplementary material for: Assessing the landscape of initiatives to improve CKD early diagnosis and treatment
Source: BMC Nephrol. 2025 Dec 12;27:50. doi: 10.1186/s12882-025-04678-z (PMC12817422; doi:10.1186/s12882-025-04678-z)
Supplement: Supplementary file 4 — Supplementary Material 4 [file 12882_2025_4678_MOESM4_ESM.pdf]

Additional file 3. Initiatives addressing multiple gaps

**Initiatives filling at least two gaps**	**Gap 1**<br>**Peer-to-peer approach**	**Gap 2**<br>**Goals & metrics**	**Gap 3**<br>**Serious outcomes**	**Gap 4**<br>**T2D & hypertensive**	**Gap 5**<br>**PCP-focused**
The Pressure is On to Diagnose CKD<br>(Europe; AstraZeneca, Primary Care Diabetes Europe, European Renal Association)	■		■	■	■
CKD Early Identification & Intervention Toolkit<br>(Global; International Society of Nephrology, KDIGO, WONCA, Primary Care Diabetes Europe)	■				■
Chronic Kidney Disease Change Package<br>(USA; National Kidney Foundation)		■			■
Kidney Disease Countermeasures Study Group Initiative 2<br>(Japan; Japanese National Government)		■			■
Care Of Patients with Chronic Non-dialysis Kidney Disease in the Family Practice<br>(Germany; German Society for General Practice and Family Medicine)	■				■
"Too Little Attention?“ Renal Insufficiency in the Family Practice<br>(Germany; German General Practitioners’ Association, German Society for General Practice and Family Medicine, Hamburg Medical Association, Young General Practice Germany)	■				■

Green fill indicates gap addressed by the initiative.

CKD, chronic kidney disease; KDIGO, Kidney Disease: Improving Global Outcomes; WONCA, World Organization of Family Doctors.
